# Temporal change in the association between life satisfaction and functional decline with gender differences: an age-specific prospective cohort study

**DOI:** 10.1265/ehpm.23-00019

**Published:** 2023-07-05

**Authors:** Naoko Shinohara, Wenjing Zhao, Yifan Shan, Shigekazu Ukawa, Hideki Ohira, Takashi Kawamura, Satoe Okabayashi, Kenji Wakai, Masahiko Ando, Kazuyo Tsushita, Akiko Tamakoshi

**Affiliations:** 1 Department of Public Health, Graduate School of Medicine, Hokkaido University, Sapporo, Japan; 2 Department of Public Health, Faculty of Medicine, Hokkaido University, Sapporo, Japan; 3 School of Public Health and Emergency Management, Southern University of Science and Technology, Guangdong, China; 4 The First Affiliated Hospital of Zhengzhou University, Zhengzhou, China; 5 Graduate School of Human Life and Ecology, Osaka Metropolitan University, Osaka, Japan; 6 Department of Psychology, Graduate School of Informatics, Nagoya University, Nagoya, Japan; 7 Kyoto University Health Service, Kyoto University, Kyoto, Japan; 8 Department of Preventive Medicine, Nagoya University Graduate School of Medicine, Nagoya, Japan; 9 Center for Advanced Medicine and Clinical Research, Nagoya University Hospital, Nagoya, Japan; 10 Comprehensive Health Science Center, Aichi Health Promotion Public Interest Foundation, Chita, Japan; 11 Faculty of Nutrition, Kagawa Nutrition University, Sakado, Japan

**Keywords:** Aged, Life satisfaction, Well-being, Functional decline, Mortality, Japan

## Abstract

**Background:**

Although life satisfaction (LS) has been shown to predict mortality, research studying the relationship between LS and functional decline is scarce. This study examined the association between LS and functional decline across four time points in young-old Japanese adults.

**Methods:**

We analysed 1,899 community-dwelling 65-year-olds in this age-specific cohort study conducted between 2000 and 2005. The Life Satisfaction Index K was used to evaluate LS and was classified into quartiles. Functional decline was determined using the Japanese Long-Term Care Insurance (LTCI) system: 1) mild disability; 2) severe disability; 3) all-cause mortality; 4) mild or severe disability; 5) severe disability or death; 6) mild or severe disability, or death. Adjusted hazard ratios (HR) with 95% confidence intervals (CI) were calculated using the Cox proportional hazard model. The analyses were conducted in the 8^th^, 10^th^, 12^th^, and 14^th^ years to assess the effect of LS on functional decline across time points.

**Results:**

The impact of LS gradually weakened over time. In the 8^th^ year (aged 72–73), a higher LS was associated with a lower risk of mild or severe disability among the women participants (adjusted HR [95% CI] = 0.30 [0.11–0.81]). However, the effect disappeared gradually (adjusted HR [95% CI] = 0.55 [0.27–1.14]) in the 10^th^ year (aged 74–75), 0.72 (0.41–1.26) in the 12^th^ year (aged 76–77), and 0.68 (0.41–1.14) in the 14^th^ year (aged 78–79). This trend continued in severe disability or death (adjusted HR [95% CI] = 0.24 [0.06–0.70], 0.31 [0.11–0.76], 0.57 [0.28–1.14], and 0.60 [0.32–1.12]) and mild or severe disability, or death (adjusted HR [95% CI] = 0.30 [0.14–0.68], 0.46 [0.24–0.87], 0.67 [0.41–1.10], and 0.65 [0.42–1.02]) in the 8^th^, 10^th^, 12^th^, and 14^th^ years, respectively. No statistically significant association was found among men at any time points or in any classification of outcomes.

**Conclusions:**

Higher LS scores in 65-year-old women were associated with a lower risk for functional decline in any combination of mild disability, severe disability, or death. Additionally, the effect of LS was observed to weaken over time.

**Trial registration:**

This is not an intervention survey and does not require registration.

## Background

Globally, the rate of ageing has increased [1]. Japan has the highest rate of ageing worldwide, with 28.4% of older adults aged ≥65 in 2019 [2]. However, it has the second highest average and healthy life expectancies globally [3]. Because of the difference between their average and healthy life expectancies, older Japanese experience functional disabilities for an average of 8.84 years in men and 12.35 years in women [2]. Functional decline has been found to be caused by cardiopulmonary diseases, neurological conditions, diabetes mellitus, cancer, obesity, dementia, affective disorders, ophthalmologic and auditory disorders, and fractures [4]. Additionally, social and financial support and physical environment have been shown affect functional decline [4].

Positive psychology, initially investigating the behavioural actions leading to well-being for all ages, has received attention in the field of psychology for several decades [5]. Meanwhile, well-being has been considered an essential factor for health-related outcomes in the ageing population [6]. Previous studies have used various tools to evaluate older people’s subjective well-being. These metrics include life satisfaction (LS), hedonic well-being, and eudemonic well-being [7] and have been linked to mortality among the geriatric population [8–11]. LS represents the evaluation of their life spans from the past to the present; hedonic well-being represents current feelings, including positive and negative ones; eudemonic well-being represents individuals’ perspectives ranging from the present to the future [5]. Many studies have demonstrated an association between functional decline and both hedonic [12–14] and eudemonic well-being [15–18]; however, studies regarding the association between functional decline and LS are limited. Although ageing does not always worsen one’s view of life [19], older people may experience the ageing process through events such as retirement, deterioration of health, and the loss of a spouse [20], which can affect LS. Previous studies have reported that older people show a decrease in LS over time [21–23]. However, the subsequent impact of LS on functional decline was unknown. Since LS was described as a variable indicating an evaluation of life mainly in the past, its effect may gradually weaken. However, to the best of our knowledge, there are no studies concerning the chronological effects of LS and its functional decline among older adults. Furthermore, women are more likely to have a disability than men. Few studies have examined the impact of LS on disability according to gender, although social determinants such as education and economic status may be responsible for this difference [24].

Regarding the effect of LS on functional decline, a cross-sectional study from Poland reported the association between LS and the prevalence of activities of daily living (ADL) and/or instrumental activities of daily living (IADL) disabilities in older inhabitants [25]. Furthermore, a prospective cohort study demonstrated that poor LS was among the strongest risk factors for declining IADL in older Norwegians [26]. Previously, we reported that low LS for 64-year-old women was a significant risk factor for the decline of the Tokyo Metropolitan Institute of Gerontology index of competence (TMIG-IC) after six years [27]. The ADL mentioned above, the IADL or TMIG-IC, merely demonstrated a single aspect of functional decline. Regarding the effect of LS on mortality, a previous study reported Finnish men aged 18–64 with high LS had a significantly lower mortality rate than women aged 18–64 with high LS [28]. The multinational World Health Organization (WHO) monitoring trends and determinants in cardiovascular disease (MONICA) project demonstrated that men aged 25–74 with higher LS have a substantial long-term survival benefit than women [29]. Further, a Korean study showed a lower hazard ratio in reducing mortality among adults 55 years of age or older [30] with high LS. These results suggest that LS affects mortality independent of age, gender, or nationality.

This study aimed to comprehensively evaluate the association between LS and functional decline using the certification of the long-term care insurance (LTCI) system across four time points to investigate a potential chronological effect of LS.

## Methods

### Study population

**Fig. 1:**
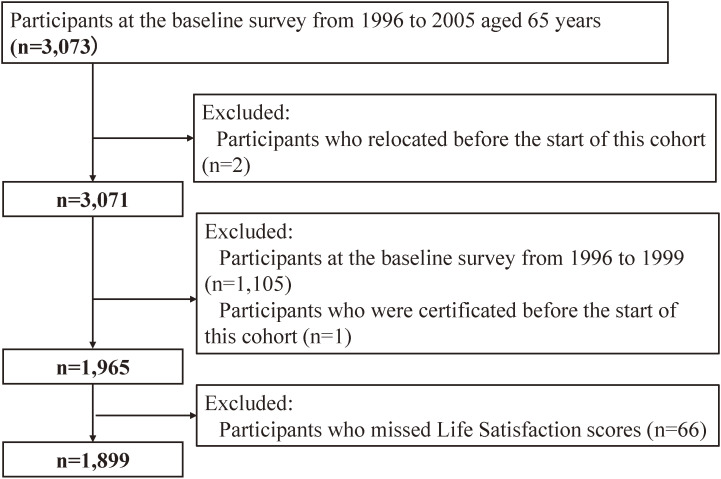
Flowchart.

The New Integrated Suburban Seniority Investigation (NISSIN) Project is an ongoing age-specific prospective cohort study [31]. The baseline investigation was conducted from 1996 through 2005 in Nisshin City, near Nagoya, Japan. It comprised 3,073 community-dwelling residents who would be 65 years old in the target year (1,548 men and 1,525 women, with an overall response rate of 43.9%). Of the 3,073 participants, two were excluded because they relocated before this cohort commenced. We excluded those who joined in 1996–1999 because they did not meet the inclusion criteria; this study focused on the LTCI system certification launched on the 1st April 2000 (n = 1,105). One participant was excluded due to the certification of long-term care before the beginning of this cohort. Additionally, we excluded the participants who had missing data regarding LS (n = 66). The final sample included 1,899 participants (977 men and 922 women), whose data were used for the analyses (Fig. 1).

This study was approved by the ethics committees of the Nagoya University Graduate School of Medicine, the National Center for Geriatrics and Gerontology of Japan, the Aichi Medical University School of Medicine, and the Hokkaido University Graduate School of Medicine. All the participants provided informed consent, with oral informed consent provided through an opt-out approach until 2001 after which written consent was provided through an opt-in approach.

### Life satisfaction

LS was evaluated using Life Satisfaction Index K (LSIK) at baseline. It is a self-administered questionnaire comprising nine items under three sections: ‘satisfaction with the whole life’, ‘evaluation of ageing’, and ‘psychological stability’ [32]. A single point was assigned for each selection of a positive option and zero points were assigned for selecting a negative option. The highest possible total score was nine, with greater total scores indicative of greater degrees of LS.

### Covariates

The covariates obtained through the self-administered questionnaire included years of participation; lifestyle variables including smoking status (never, former, current smokers), alcohol consumption (occasional, current drinkers), exercise habit (seldom, <1/week, ≥1/week); socio-demographic factors, including educational attainment (high school or lower, junior college or higher), marital status (married, other), and social activity (<10, ≥10 points). Social activities were defined as activities that required contact with society and were composed of four major facets measured using 21 questions related to professional, social, learning, and personal activities [33]. The highest possible total score was 21; higher scores indicated a higher degree of social activity. As the distribution was skewed, social activity was divided into two groups based on the median. Comorbidity variables included self-reported hypertension, diabetes mellitus, dyslipidaemia, cardiovascular disease, cerebrovascular disease, and cancer. TMIG-IC, a validated questionnaire ranging from 0–13 points, was used to access functional capacity [34]. Stress was measured by asking, ‘Do you feel stressed?’ which could be answered using three alternatives: always, sometimes, and seldom.

### Outcomes

The outcomes measured were functional decline, defined LTCI certification, and mortality. The participants were followed until relocation, received LTCI certification or they died from all causes. LTCI certification dates, deaths, and relocations were identified using the resident registry by the public health nurse in Nisshin City year-round.

Japanese residents aged ≥65 obtained long-term care certifications by applying to municipalities and receiving approval from the long-term care board that such care was needed based on their screening results [27]. The evaluation covered seven levels according to the physical or mental disorders, namely, support levels 1–2 and care levels 1–5. The higher the level of care, the more severe the functional status. At support level 1, the patient is independent with ADL with some assistance required to perform IADL; care level 5 is described as the patient requiring complete assistance to carry out ADL and being unable to function without it. Detailed information regarding the LTCI system has been described in previous studies [35–37]. Based on previous research, we divided the outcome variables into six groups: 1) mild disability (support levels or care level 1); 2) severe disability (care levels 2–5); 3) death from all-cause mortality; 4) mild or severe disability (support levels or care levels); 5) severe disability or death (care levels 2–5 or death); and 6) mild or severe disability or death (support or care levels or death). We considered care levels 2–5 to have severe functional declines because participants with care levels 1–5 had lower ADL abilities than those with support levels 1–2. For consistency over the follow-up period, the former care level 1 was divided into support level 2 and care level 1 in 2008 [36].

### Statistical analysis

**Fig. 2:**
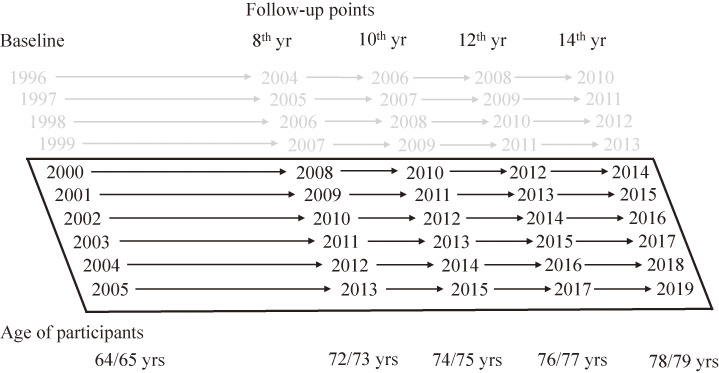
Study design. Abbreviation: yrs, years. Note: Participants from 1996 to 1999 were excluded because they did not meet the inclusion criteria, which was the LTCI system certification launched on the 1 April 2000.

LS was divided into four groups according to its distribution: 0–3, 4–5, 6, and 7–9 points. Person-years were calculated from baseline to the date of relocation, LTCI certification, death from all causes, or the end of each time point. Cox proportional hazard regression models were used to estimate the hazard ratio (HR) and the confidence interval (CI) of functional decline and mortality was set at 95%. As gender differences were found in the incidence of functional decline, we also analysed the data stratified by gender [38, 39]. The analyses were adjusted for year of participation, smoking status, alcohol consumption, exercise habits, educational attainment, marital status, social activity, comorbidity variables, functional capacity, and stress. The analyses were conducted for four time points in the 8^th^, 10^th^, 12^th^, and 14^th^ years from each baseline year to evaluate the impact of LS over time (Fig. 2). LS performed a test of trends as continuous variables. All the *p*-values evaluated were two-sided, and values less than 0.05 were considered statistically significant. Data analysis was performed using JMP Pro 14.0.0 (SAS Institute Inc., United States of America).

## Results

Tables 1 and 2 show the distributions of the baseline characteristics based on gender. Men and women with higher LS were more likely to participate in social activities, have a higher functional capacity, and experience less stress; the men had a higher level of education and a lower prevalence of hypertension and diabetes mellitus; married women exercised more frequently than married men.

**Table 1:** Association between life satisfaction and death or functional decline in men.

	**Men**								

	**Life Satisfaction (score)**								***P***

	**Q1 (0–3)**		**Q2 (4–5)**		**Q3 (6)**		**Q4 (7–9)**		
Number of participants	220		330		174		253		
Year of participation *									0.296
2000	41	(18.6)	57	(17.3)	27	(15.5)	37	(14.6)	
2001	56	(25.5)	60	(18.2)	35	(20.1)	51	(20.2)	
2002	33	(15.0)	54	(16.4)	27	(15.5)	48	(19.0)	
2003	27	(12.3)	45	(13.6)	32	(18.4)	52	(20.6)	
2004	31	(14.1)	55	(16.7)	27	(15.5)	29	(11.5)	
2005	32	(14.6)	59	(17.9)	26	(14.9)	36	(14.2)	
Educational attainment *									**0.003**
High school and lower	160	(73.1)	234	(70.9)	109	(62.6)	150	(59.3)	
Junior college and higher	59	(26.9)	96	(29.1)	65	(37.4)	103	(40.7)	
Smoking status *									0.528
Never	33	(15.0)	67	(20.3)	37	(21.3)	50	(19.8)	
Former	115	(52.3)	170	(51.5)	84	(48.3)	135	(53.6)	
Current	72	(32.7)	93	(28.2)	53	(30.5)	67	(26.6)	
Alcohol consumption *									0.688
Occasional	76	(34.7)	102	(30.9)	51	(29.3)	79	(31.2)	
Current	143	(65.3)	228	(69.1)	123	(70.7)	174	(68.8)	
Exercise habit *									0.062
Seldom	99	(45.2)	124	(37.6)	54	(31.0)	82	(32.4)	
<1/week	23	(10.5)	34	(10.3)	20	(11.5)	33	(13.0)	
≥1/week	97	(44.3)	172	(52.1)	100	(57.5)	138	(54.6)	
Marital status *									0.477
Never, widowed, or divorced	14	(6.4)	12	(3.6)	7	(4.0)	10	(4.0)	
Married	205	(93.6)	318	(96.4)	167	(96.0)	243	(96.1)	
Hypertension *									**0.046**
No	156	(70.9)	244	(73.9)	120	(69.0)	202	(79.8)	
Yes	64	(29.1)	86	(26.1)	54	(31.0)	51	(20.2)	
Diabetes mellitus *									**0.044**
No	188	(85.5)	300	(90.9)	151	(86.8)	234	(92.5)	
Yes	32	(14.6)	30	(9.1)	23	(13.2)	19	(7.5)	
Dyslipidemia *									0.562
No	194	(88.2)	303	(91.8)	157	(90.2)	227	(89.7)	
Yes	26	(11.8)	27	(8.2)	17	(9.8)	26	(10.3)	
Cardiovascular disease *									0.198
No	183	(83.2)	287	(87.0)	157	(90.2)	223	(88.1)	
Yes	37	(16.8)	43	(13.0)	17	(9.8)	30	(11.9)	
Cerebrovascular disease *									0.217
No	208	(94.6)	316	(95.8)	161	(92.5)	245	(96.8)	
Yes	12	(5.5)	14	(4.2)	13	(7.5)	8	(3.2)	
Cancer *									0.643
No	212	(96.4)	320	(97.0)	171	(98.3)	244	(96.4)	
Yes	8	(3.6)	10	(3.0)	3	(1.7)	9	(3.6)	
Social activity *									**0.004**
<10	126	(57.5)	157	(48.0)	71	(41.5)	106	(42.9)	
≥10	93	(42.5)	170	(52.0)	100	(58.5)	141	(57.1)	
TMIG-IC ^†,‡^	12	(10, 13)	12	(11.8, 13)	13	(12, 13)	13	(11, 13)	**<0.001**
Stress *									**<0.001**
Always	29	(13.2)	11	(3.4)	5	(2.9)	3	(1.2)	
Sometimes	129	(58.6)	167	(51.1)	65	(37.4)	68	(26.9)	
Seldom	62	(28.2)	149	(45.6)	104	(59.8)	182	(71.9)	

* N (%) with Chi-square test

† Median (lower quartile, upper quartile) with Kruskal-Wallis test

‡ Tokyo Metropolitan Institute of Gerontology Index of Competence score

**Table 2:** Association between life satisfaction and death or functional decline in women.

	**Women**								

	**Life Satisfaction (score)**								***P***

	**Q1 (0–3)**		**Q2 (4–5)**		**Q3 (6)**		**Q4 (7–9)**		
Number of participants	217		279		160		266		
Year of participation *									0.707
2000	48	(22.1)	48	(17.2)	26	(16.3)	34	(12.8)	
2001	35	(16.1)	49	(17.6)	26	(16.3)	41	(15.4)	
2002	31	(14.3)	47	(16.8)	23	(14.4)	49	(18.4)	
2003	38	(17.5)	47	(16.8)	33	(20.6)	52	(19.5)	
2004	35	(16.1)	41	(14.7)	25	(15.6)	39	(14.7)	
2005	30	(13.8)	47	(16.8)	27	(16.9)	51	(19.2)	
Educational attainment *									0.057
High school and lower	190	(87.6)	230	(82.4)	131	(81.9)	207	(78.1)	
Junior college and higher	27	(12.4)	49	(17.6)	29	(18.1)	58	(21.9)	
Smoking status *									0.293
Never	198	(91.2)	246	(88.2)	152	(95.0)	244	(91.7)	
Former	11	(5.1)	22	(7.9)	4	(2.5)	13	(4.9)	
Current	8	(3.7)	11	(3.9)	4	(2.5)	9	(3.4)	
Alcohol consumption *									0.677
Occasional	174	(80.2)	216	(77.4)	131	(81.9)	208	(78.2)	
Current	43	(19.8)	63	(22.6)	29	(18.1)	58	(21.8)	
Exercise habit *									**0.048**
Seldom	102	(47.0)	108	(38.7)	70	(43.8)	89	(33.5)	
<1/week	19	(8.8)	29	(10.4)	10	(6.3)	23	(8.7)	
≥1/week	96	(44.2)	142	(50.9)	80	(50.0)	154	(57.9)	
Marital status *									**0.008**
Never, widowed, or divorced	49	(22.7)	43	(15.5)	20	(12.6)	31	(11.7)	
Married	167	(77.3)	235	(85.5)	139	(87.4)	235	(88.4)	
Hypertension *									0.230
No	170	(78.3)	212	(76.0)	117	(73.1)	216	(81.2)	
Yes	47	(21.7)	67	(24.0)	43	(26.9)	50	(18.8)	
Diabetes mellitus *									0.532
No	205	(94.5)	266	(95.3)	149	(93.1)	256	(96.2)	
Yes	12	(5.5)	13	(4.7)	11	(6.9)	10	(3.8)	
Dyslipidemia *									0.168
No	179	(82.5)	219	(78.5)	139	(86.9)	219	(82.3)	
Yes	38	(17.5)	60	(21.5)	21	(13.1)	47	(17.7)	
Cardiovascular disease *									0.066
No	194	(89.4)	264	(94.6)	141	(88.1)	243	(91.4)	
Yes	23	(10.6)	15	(5.4)	19	(11.9)	23	(8.7)	
Cerebrovascular disease *									0.319
No	208	(95.9)	274	(98.2)	153	(95.6)	256	(96.2)	
Yes	9	(4.2)	5	(1.8)	7	(4.4)	10	(3.8)	
Cancer *									0.627
No	204	(94.0)	265	(95.0)	149	(93.1)	255	(95.9)	
Yes	13	(6.0)	14	(5.0)	11	(6.9)	11	(4.1)	
Social activity *									**<0.001**
<10	117	(54.2)	131	(47.1)	63	(39.9)	82	(31.2)	
≥10	99	(45.8)	147	(52.9)	95	(60.1)	181	(68.8)	
TMIG-IC ^†,‡^	13	(11, 13)	13	(12, 13)	13	(12, 13)	13	(13, 13)	**<0.001**
Stress *									**<0.001**
Always	47	(21.7)	23	(8.3)	8	(5.0)	6	(2.3)	
Sometimes	131	(60.4)	193	(69.4)	96	(60.4)	102	(38.4)	
Seldom	39	(18.0)	62	(22.3)	55	(34.6)	158	(59.4)	

* N (%) with Chi-square test

† Median (lower quartile, upper quartile) with Kruskal-Wallis test

‡ Tokyo Metropolitan Institute of Gerontology Index of Competence score

Tables 3 and 4 show the relationship between LS, long-term care certification, and death stratified by gender. The events gradually increased among men and women at each point of the 8^th^, 10^th^, 12^th^, and 14^th^ years. The women in the higher LS groups were prone to have lower risks of LTCI certification in all the categories than those in the lowest LS groups in all four time points. The second highest LS group was significantly associated with a lower risk of mild or severe disability compared to the lowest LS group. However, this trend showed a yearly U-shape. Across the four time points, the effect of LS among the second highest group was observed weakening over time in women (adjusted HR [95% CI] = 0.30 [0.11–0.81] in the 8^th^ year [aged 72–73], 0.55 [0.27–1.14] in the 10^th^ year [aged 74–75], 0.72 [0.41–1.26] in the 12^th^ year [aged 76–77], and 0.68 [0.41–1.14] in the 14^th^ year [aged 78–79]). This trend remained in severe disability or death (adjusted HR [95% CI] = 0.24 [0.06–0.70], 0.31 [0.11–0.76], 0.57 [0.28–1.14], and 0.60 [0.32–1.12]) and mild or severe disability or death (adjusted HR [95% CI] = 0.30 [0.14–0.68], 0.46 [0.24–0.87], 0.67 [0.41–1.10], and 0.65 [0.42–1.02]) in the 8^th^, 10^th^, 12^th^, and 14^th^ year, respectively. However, no associations were observed between LS and functional decline or mortality in men.

**Table 3:** Association between life satisfaction and death or functional decline in men. *

**Variables**	**Men (n = 977)**																

	**Life satisfaction ^‡^**	**Person-years**	**Number ** **of events**	**8th year^§^**		**Person-years**	**Number ** **of events**	**10th year^§^**		**Person-years**	**Number ** **of events**	**12th year^§^**		**Person-years**	**Number ** **of events**	**14th year^§^**	
				**HR (95% CI)**				**HR (95% CI)**				**HR (95% CI)**				**HR (95% CI)**	
Mild disability^†^	Q1	1723	6	1.00	(reference)	2074	9	1.00	(reference)	2395	18	1.00	(reference)	2691	26	1.00	(reference)
	Q2	2648	5	0.68	(0.18–2.56)	3215	9	0.77	(0.28–2.16)	3748	19	0.81	(0.40–1.66)	4252	28	0.74	(0.41–1.32)
	Q3	1398	5	1.14	(0.29–4.35)	1690	8	1.13	(0.39–3.29)	1960	13	0.94	(0.42–2.10)	2196	22	1.05	(0.56–1.97)
	Q4	2063	8	1.24	(0.37–4.59)	2488	10	0.91	(0.32–2.65)	2892	15	0.71	(0.32–1.57)	3271	20	0.61	(0.31–1.17)
	*P* for trend			0.573				0.867				0.409				0.108	
Severe disability	Q1	1735	6	1.00	(reference)	2084	10	1.00	(reference)	2408	18	1.00	(reference)	2705	27	1.00	(reference)
	Q2	2639	8	0.85	(0.28–2.77)	3209	11	0.72	(0.29–1.83)	3752	19	0.85	(0.42–1.74)	4268	23	0.61	(0.33–1.12)
	Q3	1404	2	0.36	(0.05–1.68)	1700	5	0.53	(0.16–1.61)	1974	10	0.82	(0.34–1.89)	2232	17	0.80	(0.40–1.55)
	Q4	2065	7	0.80	(0.24–2.82)	2495	13	0.91	(0.37–2.37)	2900	15	0.82	(0.37–1.82)	3280	22	0.65	(0.34–1.25)
	*P* for trend			0.691				0.738				0.538				0.271	
Death	Q1	1738	21	1.00	(reference)	2101	25	1.00	(reference)	2443	33	1.00	(reference)	2769	39	1.00	(reference)
	Q2	2656	16	0.67	(0.33–1.37)	3236	26	0.81	(0.44–1.47)	3794	35	0.80	(0.48–1.34)	4335	43	0.80	(0.50–1.28)
	Q3	1410	11	0.83	(0.37–1.80)	1713	17	1.01	(0.51–1.95)	1999	25	1.11	(0.63–1.93)	2266	32	1.16	(0.70–1.92)
	Q4	2078	16	0.85	(0.40–1.79)	2519	28	1.18	(0.64–2.20)	2940	33	1.02	(0.59–1.76)	3342	46	1.15	(0.71–1.87)
	*P* for trend			0.712				0.487				0.828				0.702	
Mild or severe disability	Q1	1720	11	1.00	(reference)	2061	16	1.00	(reference)	2370	30	1.00	(reference)	2650	41	1.00	(reference)
	Q2	2631	12	0.84	(0.35–2.06)	3191	18	0.78	(0.38–1.60)	3712	34	0.86	(0.50–1.47)	4199	44	0.72	(0.46–1.14)
	Q3	1392	7	0.83	(0.30–2.34)	1678	12	0.87	(0.39–1.96)	1937	20	0.90	(0.48–1.68)	2168	32	0.97	(0.58–1.60)
	Q4	2052	13	1.02	(0.41–2.58)	2471	18	0.84	(0.39–1.80)	2863	24	0.70	(0.38–1.29)	3228	33	0.62	(0.37–1.04)
	*P* for trend			0.990				0.561				0.227				0.066	
Severe disability or death	Q1	1735	25	1.00	(reference)	2084	32	1.00	(reference)	2408	46	1.00	(reference)	2705	58	1.00	(reference)
	Q2	2639	22	0.73	(0.39–1.37)	3209	32	0.73	(0.43–1.25)	3752	47	0.80	(0.52–1.25)	4268	55	0.70	(0.47–1.05)
	Q3	1404	13	0.76	(0.36–1.54)	1700	21	0.90	(0.49–1.62)	1974	31	1.03	(0.63–1.69)	2232	42	1.04	(0.67–1.60)
	Q4	2065	21	0.84	(0.43–1.64)	2495	36	1.07	(0.62–1.85)	2900	41	0.95	(0.59–1.53)	3280	59	0.99	(0.65–1.51)
	*P* for trend			0.574				0.892				0.749				0.940	
Mild or severe disability, or death	Q1	1720	29	1.00	(reference)	2061	36	1.00	(reference)	2370	56	1.00	(reference)	2650	69	1.00	(reference)
	Q2	2631	26	0.75	(0.42–1.34)	3191	39	0.79	(0.48–1.29)	3712	61	0.83	(0.56–1.24)	4199	74	0.77	(0.54–1.10)
	Q3	1392	18	0.91	(0.48–1.73)	1678	28	1.05	(0.62–1.79)	1937	41	1.06	(0.69–1.65)	2168	57	1.15	(0.79–1.68)
	Q4	2052	27	0.94	(0.51–1.71)	2471	41	1.03	(0.62–1.72)	2863	50	0.87	(0.56–1.34)	3228	70	0.93	(0.64–1.36)
	*P* for trend			0.744				0.970				0.519				0.624	

* Cox hazard models were used for calculating HRs and 95%CIs.

^†^ Mild disability concludes support levels or care level 1; severe disability concludes care levels 2–5.

^‡^ Life satisfaction (0–3, 4–5, 6, 7–9) (mean ± SD = 5.1 ± 2.0)

^§^ Model, adjusted for year of participation (2000–2002, 2003–2005), smoking status (never, former, current, or missing), alcohol consumption (occasional, current, or missing), exercise habit (seldom, less than once a week, more than once a week, or missing), educational attainment (high school or lower, junior college or higher, or missing), marital status (married, other [single, divorced, widowed], missing), social activity (<10, 10, or missing), comorbidity variable (binary; yes or no) (hypertension, diabetes, dyslipidemia, cardiovascular disease, cerebrovascular disease, cancer), Tokyo Metropolitan Institute of Gerontology Index of Competence (continuous variable), stress (always, sometimes, seldom, or missing)

Abbreviation: HR, hazard ratio; CI, confidence interval

**Table 4:** Association between life satisfaction and death or functional decline in women. *

**Variables**	**Women (n = 922)**																

	**Life satisfaction ^‡^**	**Person-years**	**Number ** **of events**	**8th year^§^**		**Person-years**	**Number ** **of events**	**10th year^§^**		**Person-years**	**Number ** **of events**	**12th year^§^**		**Person-years**	**Number ** **of events**	**14th year^§^**	
				**HR (95% CI)**				**HR (95% CI)**				**HR (95% CI)**				**HR (95% CI)**	
Mild disability^†^	Q1	1686	18	1.00	(reference)	2034	23	1.00	(reference)	2360	30	1.00	(reference)	2661	40	1.00	(reference)
	Q2	2268	13	0.58	(0.27–1.19)	2740	23	0.86	(0.47–1.57)	3181	28	0.80	(0.47–1.36)	3593	38	0.79	(0.50–1.25)
	Q3	1324	5	0.40	(0.13–1.03)	1608	10	0.65	(0.29–1.36)	1870	19	0.93	(0.50–1.68)	2111	22	0.80	(0.46–1.36)
	Q4	2166	9	0.46	(0.18–1.10)	2633	16	0.71	(0.34–1.44)	3080	25	0.82	(0.45–1.48)	3493	37	0.90	(0.54–1.49)
	*P* for trend			0.041				0.272				0.857				0.749	
Severe disability	Q1	1748	6	1.00	(reference)	2116	8	1.00	(reference)	2471	12	1.00	(reference)	2810	14	1.00	(reference)
	Q2	2275	8	0.94	(0.31–3.01)	2766	8	0.68	(0.24–1.93)	3241	12	0.70	(0.30–1.63)	3693	17	0.90	(0.44–1.90)
	Q3	1334	1	0.21	(0.01–1.30)	1627	3	0.43	(0.09–1.56)	1909	7	0.69	(0.25–1.81)	2174	9	0.81	(0.33–1.90)
	Q4	2191	5	0.47	(0.12–1.81)	2670	11	0.68	(0.24–2.00)	3133	15	0.70	(0.30–1.67)	3582	15	0.68	(0.30–1.54)
	*P* for trend			0.115				0.242				0.256				0.200	
Death	Q1	1760	10	1.00	(reference)	2142	12	1.00	(reference)	2518	14	1.00	(reference)	2878	20	1.00	(reference)
	Q2	2302	9	0.52	(0.20–1.34)	2807	10	0.47	(0.19–1.13)	3298	17	0.72	(0.35–1.51)	3766	23	0.74	(0.40–1.39)
	Q3	1334	4	0.34	(0.08–1.13)	1630	4	0.29	(0.07–0.90)	1919	7	0.49	(0.18–1.23)	2198	8	0.42	(0.17–0.96)
	Q4	2196	8	0.46	(0.16–1.30)	2688	13	0.62	(0.26–1.50)	3168	17	0.70	(0.32–1.54)	3638	21	0.63	(0.32–1.25)
	*P* for trend			0.069				0.243				0.373				0.276	
Mild or severe disability	Q1	1674	22	1.00	(reference)	2014	27	1.00	(reference)	2325	38	1.00	(reference)	2615	48	1.00	(reference)
	Q2	2249	19	0.67	(0.36–1.25)	2711	29	0.86	(0.50–1.48)	3143	35	0.75	(0.47–1.20)	3542	47	0.78	(0.52–1.18)
	Q3	1324	5	0.30	(0.11–0.81)	1607	11	0.55	(0.27–1.14)	1866	20	0.72	(0.41–1.26)	2104	24	0.68	(0.41–1.14)
	Q4	2161	13	0.46	(0.21–0.98)	2621	22	0.69	(0.36–1.29)	3057	34	0.76	(0.45–1.29)	3456	46	0.84	(0.53–1.32)
	*P* for trend			0.010				0.109				0.383				0.346	
Severe disability or death	Q1	1748	16	1.00	(reference)	2116	19	1.00	(reference)	2471	24	1.00	(reference)	2810	31	1.00	(reference)
	Q2	2275	16	0.62	(0.30–1.28)	2766	17	0.53	(0.27–1.05)	3241	25	0.66	(0.37–1.18)	3693	35	0.78	(0.47–1.29)
	Q3	1334	4	0.24	(0.06–0.70)	1627	6	0.31	(0.11–0.76)	1909	13	0.57	(0.28–1.14)	2174	16	0.60	(0.32–1.12)
	Q4	2191	12	0.43	(0.19–1.00)	2670	20	0.53	(0.26–1.09)	3133	27	0.63	(0.34–1.16)	3582	30	0.61	(0.35–1.06)
	*P* for trend			0.014				0.041				0.105				0.082	
Mild or severe disability, or death	Q1	1674	31	1.00	(reference)	2014	37	1.00	(reference)	2325	49	1.00	(reference)	2615	61	1.00	(reference)
	Q2	2249	27	0.61	(0.36–1.04)	2711	38	0.74	(0.46–1.19)	3143	48	0.73	(0.49–1.10)	3542	65	0.80	(0.56–1.14)
	Q3	1324	8	0.30	(0.14–0.68)	1607	14	0.46	(0.24–0.87)	1866	26	0.67	(0.41–1.10)	2104	31	0.65	(0.42–1.02)
	Q4	2161	20	0.45	(0.24–0.84)	2621	30	0.60	(0.35–1.02)	3057	44	0.69	(0.44–1.08)	3456	59	0.77	(0.52–1.15)
	*P* for trend			0.002				0.022				0.153				0.189	

* Cox hazard models were used for calculating HRs and 95%CIs.

^†^ Mild disability concludes support levels or care level 1; severe disability concludes care levels 2–5.

^‡^ Life satisfaction (0–3, 4–5, 6, 7–9) (mean ± SD = 5.2 ± 2.1)

^§^ Model, adjusted for year of participation (2000–2002, 2003–2005), smoking status (never, former, current, or missing), alcohol consumption (occasional, current, or missing), exercise habit (seldom, less than once a week, more than once a week, or missing), educational attainment (high school or lower, junior college or higher, or missing), marital status (married, other [single, divorced, widowed], missing), social activity (<10, 10, or missing), comorbidity variable (binary; yes or no) (hypertension, diabetes, dyslipidemia, cardiovascular disease, cerebrovascular disease, cancer), Tokyo Metropolitan Institute of Gerontology Index of Competence (continuous variable), stress (always, sometimes, seldom, or missing)

Abbreviation: HR, hazard ratio; CI, confidence interval

## Discussion

Herein, we found that high LS was associated with a lower risk of functional decline based on the LTCI system; however, the impact of LS weakened gradually over time among women while no such association was reported among men. This was the first study of an age-specific cohort to demonstrate LS’s chronological impact on classified functional decline among the young and old populations stratified by gender. Although our previous study investigated LS and functional decline in gender stratification, its results were limited due to a single cut-off point for functional decline [27]. It did not follow the association between functional decline and the chronological impact of LS. The findings showed that LS could be an indicator expressing the risk of functional decline regardless of mild disability or severe disability for women; furthermore, the impact of LS may be gradually weakened among women.

Some previous studies have explored the relationship between social support, economic status, health, and LS [40, 41]. LS reflects many aspects of people’s lives and vice versa. High LS may improve their lives and prevent older people from worsened health conditions and dying.

Among women, the effect of LS on functional decline diminished over time. LS is known to reflect an individual’s well-being from the past to the present [5]. The effect of showing well-being may weaken over time as it gets further from the baseline. Furthermore, older adults find it difficult to have the opportunities and resources to live positively because of their decreasing life span. They feel less motivated moving forward while focusing on maintaining health and preventing losses [22]. In a 15-year longitudinal study, more decline in social participation over time and less perceived support at the end of life indicated a more severe terminal decline in LS among older people [21]. Consequently, the effect of LS may decrease the impact of preventing functional decline over time.

We found that the second highest level of LS is likely lower the subsequent risk of functional decline and subsequent death in women by 70% in the 8^th^ year. Women tend to live longer on average and have a lower risk of suffering from fatal diseases than men; conversely, older women suffer from higher rates of functional disability than men [42]. One possible mechanism that prevents functional decline in women with high LS is gender difference in social roles. The global concept of gender roles is based on the division of labour in the family [43]. Traditionally, women perform more housework, cooking, and nurturing than men. Therefore, women can prepare meals to maintain their well-being, leading to healthier diets than men [44]. Research has positively linked LS with good nutrition [45]. We observed differences in our participants’ food intake based on gender; although women consumed more vegetables and fruits than men, men ate more rice than women (data not shown). Previous studies reported that when older adults actively support their families, such as by frequently caring for grandchildren, their LS is positively influenced [46]. These household responsibilities are considered the primary preventive factors for maintaining LS by women in Japan [47], thus leading to this result.

Moreover, women may use social networks well. In our study, participants with higher LS indicated higher social activities. Higher LS led older people to participate in social activities, subsequently increasing their physical and mental activities, which are protective factors for disability [48]. Having many friends and engaging in many social activities enhanced women’s LS; however, men were more satisfied with life when living with their spouses [49].

Despite not being stratified by gender, our results were consistent with those reported in previous studies that demonstrated a protective effect of LS on functional decline in Norwegian adults aged 60–69 [26] and Polish adults aged 71–80 [25]. Our age-specific cohort research design avoided the wide age range and showed the benefits of LS for subsequent functional decline in young and old women. Furthermore, the tools employed in previous studies, TMIG-IC [27], ADL [25, 26], and IADL [26], can evaluate older people’s functional ability. In our study, by contrast, we defined the Japanese LTCI system and assessed it comprehensively using classified information that covered physical or mental disorders (support levels 1–2 and care levels 1–5). We divided the data into six groups and analysed it to determine the association between LS and functional decline and to follow four time points to show the chronological effect of LS. Higher LS scores in women were associated with a lower risk for functional decline in any combination of mild disability, severe disability, or death, with the effect of LS weakening over time across the four time points.

In this sophisticated society, our study provides evidence for establishing a healthy ageing community to decrease the risk of functional decline besides the traditional risk factors by maintaining a higher LS. From a health behaviour perspective, lower LS is likely to impact its chronological effect and further lead to functional decline or mortality. LS is positively associated with health behaviours—such as not smoking, engaging in physical exercise, using sun protection, eating fruits, and limiting fat intake—known as protective factors for well-being [50].

This study’s strengths included the following: first, we targeted participants who attained 65 years old at the beginning, (i.e., the onset of old age), as the incident rate of disability has risen exponentially with age, and followed up on this population. These results allowed us to eliminate the confounding factor of age. Second, our study enabled the examination of LS by assessing functional decline in detail according to the classification of the Japanese LTCI, which in previous studies was measured using only a single criterion.

Despite the strengths, there were some limitations associated with our study. First, the statistical power may not be sufficient due to the relatively small sample size, low functional decline, and death incident rate. Nisshin, the city where this research was conducted, is located in the Aichi prefecture, which has a higher healthy life expectancy than other prefectures [51]. Thus, this sample may have resulted in lower incidence of functional decline. Second, the LTCI system provided in Japan is a unique healthcare service; therefore, we cannot directly generalise this study’s results to other parts of the world. There are specific steps involved in the registration of functional decline by LTCI: apply, examine, and record [52]. This process must be carried out first by the older adults, their families, or care managers. However, because of the traditional Japanese concept of older adult care, which states that older people should be tended to by their families [53–55]. Unreported cases, those who are cared for by their families but not reported to the public office, may cause underestimation. Third, we could not follow relocated people, which were recorded as lost to follow-up. Although the number of relocated people was small (n = 142, 7.5%), we found that the relocated people had significantly lower LS scores than the participants. It could lead to an underestimation of the study’s results.

## Conclusions

In conclusion, our findings suggested that LS might be an indicator measuring the risk of functional decline, especially during severe levels or death. At the same time, the impact of LS may decrease chronologically in Japanese young and old women. We believe the findings in this study provide new insights and useful parameters for older adults living in communities in Japan for future social medicine, hygiene, and preventive medicine.

## Abbreviations

LS: Life Satisfaction
LSIK: the Life Satisfaction Index K
LTCI: Long-Term Care Insurance
CI: Confidence Intervals
HR: Hazard Ratio
ADL: Activities of Daily Living
IADL: Instrumental Activities of Daily Living
NISSIN: the New Integrated Suburban Seniority Investigation
TMIG-IC: the Tokyo Metropolitan Institute of Gerontology Index of Competence
WHO: World Health Organization

## Declarations

### Availability of data and material

The datasets generated during and/or analysed during the current study are available from the corresponding author at a reasonable request.
